# Rapid mineralization of hydrothermally induced TiO_2_ coating on titanium and early osteogenic responses in vitro

**DOI:** 10.1007/s10856-026-07095-2

**Published:** 2026-06-16

**Authors:** Faleh Abushahba, Sherif A. Mohamad, Adrian Stiller, Polina Sinitsyna, Nagat Areid, Jan-Henrik Smått, Leena Hupa, Pekka K. Vallittu, Terhi J. Heino, Timo Närhi

**Affiliations:** 1 Department of Biomaterials Science and Turku Clinical Biomaterials Center—TCBC, Institute of Dentistry, University of Turku, Turku, Finland; 2 Department of Prosthetic Dentistry and Stomatognathic Physiology, Institute of Dentistry, University of Turku, Turku, Finland; 3 Institute of Biomedicine, Faculty of Medicine, University of Turku, Turku, Finland; 4 Johan Gadolin Process Chemistry Centre and High-Temperature Processes and Materials, Åbo Akademi University, Turku, Finland; 5 Laboratory of Molecular Science and Engineering, Åbo Akademi University, Turku, Finland; 6 The Wellbeing Service County Southwest Finland, Turku, Finland

## Abstract

This study evaluated hydrothermally induced nanostructured TiO_2_ coatings (HT-TiO_2_) on Grade 1 titanium (Ti) with a focus on surface mineralization behavior and early cellular responses. Rapid mineralization (RM) was achieved by immersion in modified simulated body fluid (m-SBF). Ti discs were subjected to hydrothermal treatment (HT) to produce a TiO_2_ coating, then mineralized in m-SBF for 2, 4, or 6 h. Surface characterization was conducted using SEM, EDX, XRD, and FTIR. Cellular responses were assessed using pre-osteoblastic cells and mesenchymal stem cell (MSC)‑like cells in vitro. Cell proliferation on Ti substrates was evaluated over 7 days, and alkaline phosphatase (ALP) activity in MSC-like cells was measured at days 7 and 14 across four groups: NC (non-coated), HT-TiO_2_, RM4 (HT-TiO_2_ with 4-h RM), and RM6 (HT-TiO_2_ with 6-h RM) discs. Cell attachment and spreading were evaluated by fluorescence microscopy at days 3 and 7. The intensity of the Ca–P layer and the molar composition increased with mineralization time (2–6 h). All surfaces supported pre-osteoblast proliferation without significant differences between groups. ALP activity was significantly elevated in the HT-TiO_2_ and RM6 groups compared to controls, indicating early osteogenic activity in a subset of cells. Fluorescence imaging showed flattened cell morphology on HT-TiO_2_-discs and more elongated morphology on RM discs at day 3, while comparable cell confluency was observed on all surfaces by day 7. Overall, hydrothermally-induced nanostructured TiO_2_ coatings on Ti surfaces can be rapidly mineralized, and the resulting Ca–P layer supports cell attachment, proliferation, and early osteogenic activity in vitro. These findings highlight the potential of surface‑driven mineralization strategies for modulating early cellular responses to titanium implants.

**Graphical Abstract:**

**Figure:**
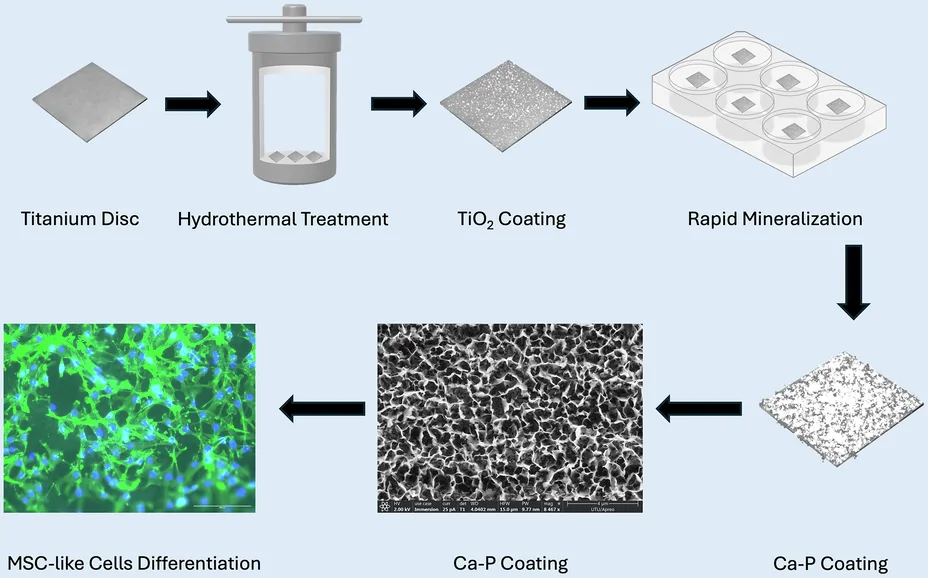

## Introduction

Dental implants are commonly used to replace missing teeth in patients with partial or complete edentulism. Titanium (Ti) is the material of choice for most oral and orthopaedic implants because of its mechanical strength, corrosion resistance, chemical stability, and excellent biocompatibility [1, 2]. Research has consistently demonstrated the good success rates of dental implant treatment [3, 4]. Continuous advancements in implant design, surface modifications, and material technology have further improved outcomes. Despite the excellent biocompatibility and good mechanical properties of Ti, osseointegration tends to occur slowly, mainly because the spontaneously formed oxide layers are biologically inert and exhibit limited interactions with newly forming bone [5]. A variety of surface modification techniques have been applied to dental implants to enhance osseointegration, such as grit blasting [6], acid etching [7], plasma spraying [8], alkali and heat treatment [9], plasma electrolytic oxidation [10], electrophoretic deposition coating [11], and hydrothermal (HT) treatment [12].

Nanostructured TiO_2_ coatings present a practical approach for introducing a bioactive layer on Ti implant surfaces. Previous studies report that the smooth, hydrothermally induced nanostructured TiO_2_ (HT-TiO_2_) coatings of oxide layer thickness of 100-200 nm [13], enhance wettability and facilitate better adhesion and proliferation of epithelial and gingival fibroblast cells, potentially improving tissue attachment on implant surfaces [14–17]. Furthermore, osteoid formation has been observed within the small bone fragments in direct contact with the HT-TiO_2_-coated surface, as demonstrated in tissue sections harvested after 14 days of culture using a pig organotypic tissue model [18]. In addition to promoting tissue integration, the HT-TiO_2_ coating has also shown the ability to inhibit bacterial adhesion and the initial biofilm formation [19]. These favorable biological responses highlight the potential of HT-TiO_2_ coatings to improve long-term implant stability by promoting soft tissue attachment, reducing the risk of peri-implantitis, and improving osseointegration, making them a promising strategy for the development of dental and orthopaedic implant surfaces.

A growing body of research shows the benefits of functionalizing implant surfaces with calcium phosphate (Ca-P) coatings. Along with enhancing osteoblast adhesion and proliferation, Ca-P coatings, such as hydroxyapatite and β-Tricalcium phosphate, have been shown to stimulate new bone formation by driving the differentiation of mesenchymal stem cells (MSC) and pre-osteoblasts into mature osteoblasts [20–22]. One approach for achieving a particularly rapid formation of Ca-P coating involves using highly concentrated Ca-P solutions. For example, a high ionic strength solution (>1100 mM), with calcium and phosphate concentrations 10 times higher than in standard simulated body fluid (SBF), enabled Ca-P deposition onto Ti alloy substrates at a linear rate within just a few hours, significantly faster than the 14 days typically required with standard SBF [23, 24].

The present study investigates how hydrothermally induced nanostructured TiO_2_ coatings (HT-TiO_2_) on Ti affect osteoblast cell behavior, including pre-osteoblast proliferation and early osteogenic activity of MSC-like cells. Furthermore, the study explores whether depositing Ca-P using rapid mineralization (RM) of HT-TiO_2_ surfaces, achieved by supersaturated modified simulated body fluid (m-SBF), can further modulate early cellular responses. The overall goal is to create a more favorable environment for bone regeneration and osseointegration.

## Materials and methods

### Titanium discs preparation

#### Polishing of titanium discs

A 25 cm² Grade 1 commercially pure Ti sheet (William Gregor Ltd., London, UK), with a thickness of 1 mm, was cut into 1 cm² discs. The discs were then polished from one side with grit silicon carbide papers in the following order: 800, 1200, 2400, and 4000. Then the Ti discs were cleaned in an ultrasonic bath with acetone for 15 min, followed by rinsing in double-distilled water and drying at room temperature.

#### Hydrothermal coating

The HT-TiO_2_ coating of Ti discs was prepared by the HT method. The HT suspension was prepared by dissolving TiO_2_ powder in Milli-Q (MQ) water and mixed for 5 min. Then, 1:10 diluted tetramethylammonium hydroxide (TMAH)(N(CH3)4^+^ OH)^−^ was added to the suspension and mixed for an additional 30 min. Ti discs were put at the base of Teflon vessels surrounded by stainless-steel jackets, and the HT suspension was poured. The vessels with the discs were then kept at 150 ± 10 °C in a constant-temperature oven for 48 h. Following the HT treatment, the Ti discs were removed from the vessel and allowed to cool at room temperature. The discs were then washed in an ultrasonic bath containing distilled water for 15 min, after which they were rinsed with acetone and ethanol (5 min each).

### Mineralization of HT-TiO_2_ discs

The chemicals given in Table 1 were used to prepare the m-SBF mineralization solution and were added in the appropriate order to a glass beaker containing 900 mL of deionized water at room temperature (RT). The next chemical was added after the previous one had completely dissolved in water, then the solution was made up to 1 L by adding the required amount of water (pH 4.4 at RT). Immediately preceding the mineralization process, NaHCO_3_ powder was added under vigorous continuous stirring to the solution to raise the HCO3- concentration to 9.5 mM (pH 6.5 at RT). The HT-TiO_2_ discs were positioned in 6-well plates (Thermo Fisher Scientific, Waltham, Massachusetts, US) with their polished side facing up. Then, 10 mL of the m-SBF was added to each well. Mineralization was allowed for 2 h (RM2), 4 h (RM4), and 6 h (RM6) at RT. Upon completion of mineralization, the discs were cleaned 3 times with deionized water, then allowed to dry at RT.

**Table 1:** Reagents, their order, and amount for the preparation of mineralization solution, for a volume of 1 L

Reagent	Order	Amount
NaCl	1	58.44 g
KCl	2	0.373 g
CaCl_2_.2H_2_O	3	3.675 g
MgCl_2_.6H_2_O	4	1.016 g
NaH2PO_4_.H_2_O	5	1.380 g

### Ion concentration measurements of the m-SBF

The elemental concentrations of Ca, Na, K, Mg, and P in m-SBF were determined by inductively coupled plasma optical emission spectrometry (ICP-OES; Optima 5300 DV, PerkinElmer, Waltham, USA). Prior to dilution, samples were acidified with concentrated HNO₃ to prevent possible precipitation. Specifically, 0.15 mL of m-SBF was mixed with 0.30 mL of HNO₃ (65 wt.%, Suprapur, Merck), followed by the addition of 10.0 mL of ultrapure water (overall dilution factor 69.7). Mean elemental concentrations were calculated from five independently prepared replicates. The ICP-OES instrument was calibrated using ultrapure water and certified single-element (P, K) and multi-element (Na, Mg, Ca) standards (Spectrascan, Teknolab AS, Norway; K standard from Ultra Scientific, Agilent Technologies, USA). Calibration curves were established using standards of 0, 1, 5, and 20 ppm for each element. Emission lines and viewing modes used for quantification were: Ca 315.887 nm (radial), K 766.490 nm (radial), Mg 285.213 nm (radial), Na 589.592 nm (radial), and P 213.617 nm (axial).

### Surface characterization

#### Scanning electron microscopy and energy dispersive X-ray analysis

Scanning electron microscopy (SEM, Leo 1530, Oberkochen, Germany) combined with energy dispersive X-ray analysis (EDX, UltraDry, Thermo Scientific, Madison, USA) was used to analyze the surface morphology and composition of the Ti discs. The analysis was conducted for (1) non-coated (NC) discs, (2) HT-TiO2-Ti discs, and (3) the experimental groups RM2, RM4, and RM6. In addition, the Ca-P coating thickness was evaluated by SEM through 10 randomly selected measurements.

#### Contact angle measurements

Contact angle (CA) values were evaluated to determine the surface wettability of the NC, HT-TiO₂, RM2, RM4, and RM6 Ti discs using the sessile drop method described by Jong et al. [25], and utilizing a Theta Optical tensiometer (CAM100, KSV Instruments Ltd., Espoo, Finland). A total of 120 images were captured for each droplet deposited on the surface over 20 s. The CA values were determined by fitting the Young–Laplace equation to the droplet shape. To improve precision, the sessile drop method was integrated with axisymmetric drop shape analysis (ADSA), which allows simultaneous estimation of both CA and surface tension. The mean CA of six independent droplets was calculated for each experimental group.

#### Surface roughness measurements

A 3D non-contact optical profilometer (Bruker Nano GmbH, Billerica, MA, USA) was used to determine the arithmetical mean heights (Sa) of the NC, HT-TiO_2_, RM2, RM4, and RM6 disc surfaces. Six randomly selected locations were measured for each sample with a 5× objective lens and a 0.5x multiplier. A back scan and length settings of 20 μm and 60 μm in VSI/VXI mode were applied, and Vision 64 software was used to calculate the Sa roughness parameters.

#### X-ray diffraction analysis

X-ray diffraction (XRD) measurements were performed on NC, HT-TiO₂, RM2, RM4, and RM6 using a Bruker D8 Discover diffractometer (Bruker AXS GmbH, Karlsruhe, Germany) equipped with a Cu Kα radiation source (λ = 1.5406 Å) and a HI-STAR area detector. The analysis was carried out in grazing incidence configuration, with a fixed incidence angle of 3° to enhance surface sensitivity. The detector scanned over a 2θ range from 10° to 60°, with a step size of 0.04° and a dwell time of 3 s per step. Data were processed using DIFFRACplus LEPTOS software (version 7.03, Bruker).

#### Fourier-transform infrared spectroscopy

Fourier-transform infrared (FTIR) spectroscopy was used to study the formation of Ca-P on the RM2, RM4, and RM6. Spectra were acquired in absorbance mode using an IFS 66S spectrometer (Bruker Optics, Ettlingen, Germany) equipped with a deuterated triglycine sulfate (DTGS) detector. Measurements were conducted at an incident angle of 45° using a variable-angle external reflection accessory (Seagull, Harrick) coupled with a zinc selenide (ZnSe) hemispherical crystal (Harrick Scientific Products). A wavenumber range of 400–4000 cm^−1^ with a resolution of 4 cm^−1^, averaging 32 scans per spectrum, was applied to collect the Spectra.

### Osteoblast proliferation and early osteogenic responses

#### Cell cultures

Mouse MC3T3-E1 pre-osteoblasts (subclone 4; ATCC, CRL-2593) and C3H10T1/2 MSC-like cells were used in the current study. Cultures were expanded in T75 flasks in phenol red-free αMEM (Gibco, UK) with 10% FBS (Gibco, UK) and 1% Penicillin–Streptomycin (Gibco, USA). The cells were grown at 37 °C, 5% CO_2_ (SANYO, Japan), and the media was changed every 2–3 days. Cells used in the experiments were 80–100% confluent and between passages 12–15 for MC3T3-E1 cells and 4–7 for C3H10T1/2 cells.

#### Pre-osteoblast proliferation

The proliferation of pre-osteoblastic MC3T3-E1 cells was evaluated on HT-TiO_2_, RM4, and RM6 discs, with NC discs serving as controls. Prior to cell seeding, sterile Ti discs were transferred to a 24-well plate and incubated in cell culture media at 37 °C, 5% CO_2_ for 15 min. Cells were seeded at a density of 25,000 cells per disc and allowed to grow for up to 7 days. On days 1, 3, and 7, the Alamar Blue (Bio-Rad, USA) assay was performed. In short, cells were incubated in 10% Alamar Blue solution at 37 °C, 5% CO_2_ for 4 h, whereafter 100 μl of each sample was transferred to a 96-well plate in duplicates. An Ensight Multimode Microplate Reader (PerkinElmer, USA) was used to measure the fluorescence excitation/emission at 560/590 nm. Afterwards, cells were washed with PBS and then cultured in fresh cell culture media until the consecutive time point. These experiments were carried out as three biological repeats with four technical samples per repeat.

#### Live/dead staining and fluorescent imaging of pre-osteoblasts

At day 7, a sample from each group was subjected to Live/Dead staining using the Cell Imaging Kit (ThermoFisher Scientific, USA). To prepare the master mix, 1 ml of the Live stain (Calcein) was mixed with 1 µL of the Dead stain (BOBO-3 Iodide) and vortexed for 10 s. Subsequently, half of the cell culture media was pipetted out of each well, and 500 µL of the staining master mix was added to the remaining medium. The plates were incubated for 15 min at RT, protected from light. Samples were viewed using the Evos 5000 Microscope (Invitrogen, USA) with the excitation/emission wavelengths of 470/525 and 531/593 for Calcein and BOBO-3 Iodide, respectively.

#### Alkaline phosphatase (ALP) activity, staining, and DNA quantification in MSC-like cells

C3H10T1/2 cells, representing a multipotent mesenchymal precursor (MSC-like) cell line, were seeded at a density of 35,000 cells per well and incubated for 24 h. Subsequently, the media was replaced with osteogenic induction medium containing 0.1 μM dexamethasone (Fisher Scientific, UK), 50 µg/ml ascorbic acid, and 10 mM β-glycerophosphate (Sigma-Aldrich, USA) [26–28].

To verify the osteogenic induction capacity, cells seeded on tissue culture plastic and cultured in either osteogenic or basal media were subjected to ALP and Von Kossa staining at day 7. These stainings were used to assess ALP activity and mineral deposition under osteogenic conditions and were not intended to evaluate substrate‑specific effects.

ALP staining was performed using a commercially available kit (Sigma-Aldrich, USA) according to the manufacturer´s instructions. Sodium nitrite and FRV alkaline solutions were mixed in a 1:1 ratio and incubated for 2 min at RT. dH_2_O and Naphtol AS-BI solutions were then mixed (45:1), and combined with the sodium nitrite/FRV solution. Cells fixed in 4% paraformaldehyde (PFA, Santa Cruz Biotechnology, The Netherlands) for 30 min were washed with PBS, incubated with the staining reagent in the dark for 15 min, followed by washing with dH_2_O and air-drying for 30 min. For von Kossa staining, cells were washed with PBS, fixed for 30 min then rinsed twice with dH_2_O. A 2% silver nitrate solution was added, and samples were incubated for 60 min under a 60 W lamp. Cells were subsequently washed three times with dH_2_O, incubated with 2.5% sodium thiosulphate, washed twice with dH_2_O, and air‑dried for 30 min. Imaging was performed using a phase contrast microscope (Olympus, Japan).

For quantitative detection of ALP activity at days 7 and 14, MSC-like cells cultures on Ti discs were washed with PBS, lysed (50 mM Tris-HCl, 0.1% Triton X-100, 0.9% NaCl), and scraped from each disc. Lysates were transferred to Eppendorf tubes, vortexed for 10 s, and stored at −80 °C until further analysis. Before performing the assay, the lysates were thawed at RT, vortexed for 10 s, and centrifuged for 15 min at 13,000 rpm for 15 min at 4 °C. ALP activity was determined using P-nitrophenylphosphate (pNPP) (Sigma-Aldrich, USA) as the substrate. Briefly, 100 μL of assay solution (15 µL of 0.1 M pNPP mixed with 85 μL of assay buffer containing 0.1 M Tris, 1 mM MgCl_2_) was pipetted into a 96-well plate in duplicate, followed by the addition of 10 μl of lysate. Samples were incubated for 1 h at RT, protected from light, after which the reaction was stopped by adding 100 μL 1 N NaOH. Absorbance eas measured at 405 nm using a microplate reader.

To normalize ALP activity to cell number, DNA quantification was performed on the same lysates used for ALP activity measurement using the Quant-iT™ dsDNA High Sensitivity (HS) Assay Kit (ThermoFisher Scientific, USA) according to the manufacturer´s instructions. Briefly, 200 µL of the working solution (dsDNA HS reagent diluted 1:200 in dsDNA HS buffer) was pipetted into a fresh 96-well plate in duplicate, followed by 10 µL of standards or samples. Plates were gently mixed and incubated for 5 min at RT, protected from light. Fluorescence was measured at excitation/emission wavelengths of 480/530 nm using a microplate reader.

ALP activity was assessed in three independent biological experiments, each performed with three technical replicates.

#### Attachment and spreading of MSC-like cells

On days 3 and 7 of cell culture, a disc from each group was washed in PBS, fixed in 4% PFA (Santa Cruz Biotechnology, The Netherlands) for 30 min, before being incubated with phalloidin (F-Actin) (Abcam, UK) (diluted 1:1000) for 20 min, protected from light. For nuclear staining, the discs were incubated in a 1:100 dilution of Hoechst 33258 (Sigma-Aldrich, USA) for 10 min, protected from light. Discs were viewed using the Evos 5000 Microscope with the excitation/emission wavelengths used of 357/447 and 470/525 for Hoechst and F-Actin, respectively.

### Statistical analysis

The differences in the proliferation rates and ALP activity among the experimental groups were evaluated using a one-way analysis of variance (ANOVA), and statistical significance was defined as *p* < 0.05. Statistical analyses were performed using the Statistical Package for the Social Sciences (SPSS), software version 29.0 (SPSS Inc., Chicago, IL, USA), and the results are shown as mean values and standard deviations (SD).

## Results

### Characterization

#### Ion concentration in the m-SBF

Table 2 shows the ion concentration in the m-SBF and their counterparts in the SBF and human blood plasma, based on Kokubo and Takadama [29], with ionic concentrations converted from mmol·L⁻¹ to mg/L. ICP-OES analysis revealed that the Ca concentration in m-SBF was slightly less than ten times higher, while the P concentration was more than eleven times higher, compared with those in Kokubo’s SBF.

**Table 2:** Elemental concentrations of Na, K, Ca, Mg, and P in the supersaturated mineralization solution (m-SBF) and compared to simulated body fluid (SBF, Kokubo and Takadama [28])

Ion	m-SBF (mg/L)	Kokubo SBF (mg/L)
Na	23,427.7 (146.6)	3,261.6
K	251.7 (10.2)	195.5
Ca	983.4 (19.7)	100.2
Mg	154.7 (2.9)	36.5
P	363.4 (2.0)	31

m-SBF concentrations were measured in this study; SBF values are from the literature [28] and were converted from mmol·L⁻¹ to mg/L. m-SBF values are reported as mean ± SD (*n* = 5).

#### SEM-EDX analysis and surface contact angle

**Fig. 1:**
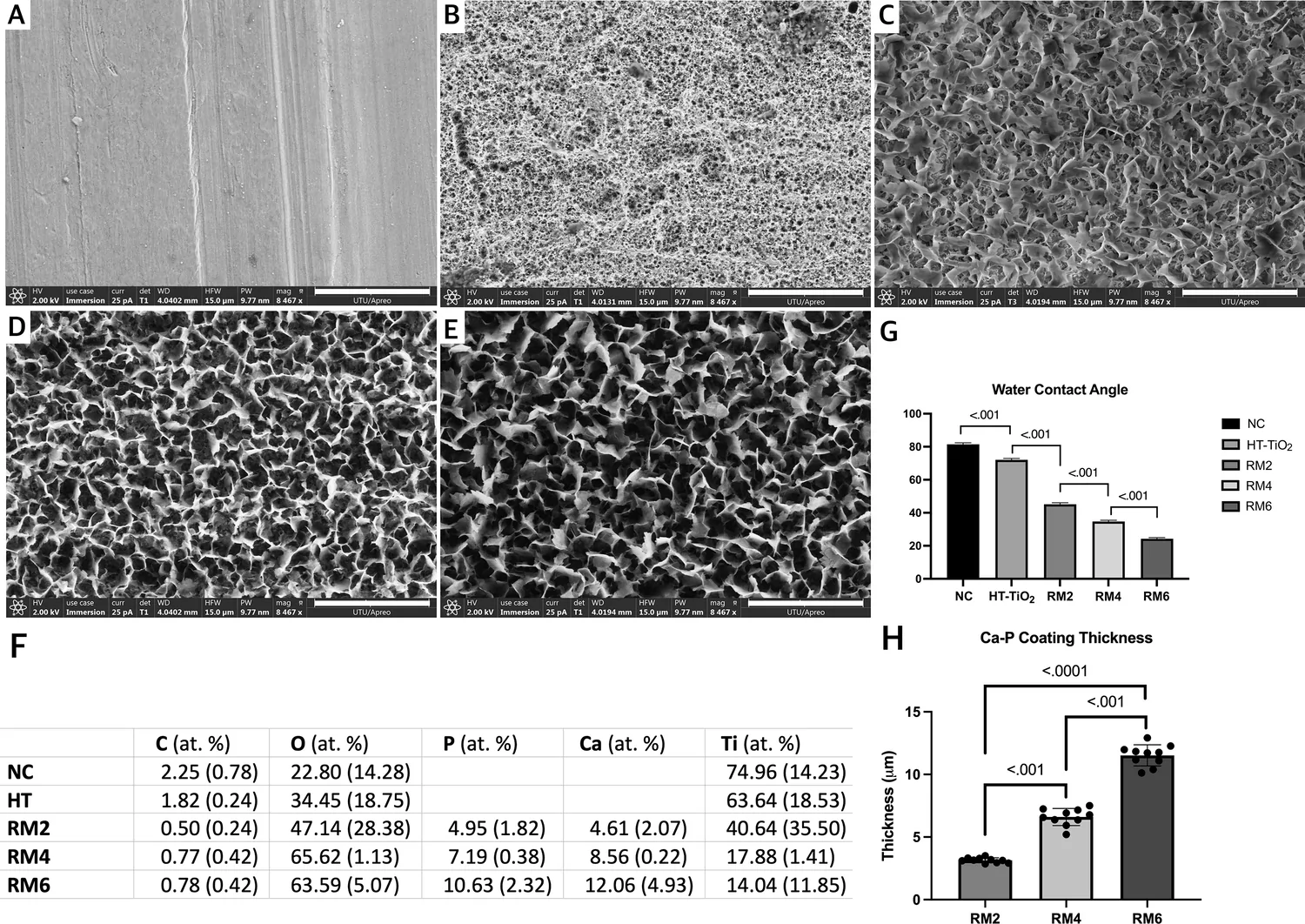
SEM images of **A** the non-coated (NC), **B** hydrothermally induced nanostructured TiO_2_ (HT-TiO2) coating, and after rapid mineralization for **C** 2 h (RM2), **D** 4 h (RM4), and **E** 6 h (RM6), scale bar = 4 µm. **F** shows the EDS analysis of elements on the mineralized discs in atom%. (*n* = 3). **G** represents water contact angle measurements on all experimental groups. **H** shows the calcium-phosphate coating thickness on RM2, RM4 and RM6. Measurements were the average of 10 randomly selected location

Figure 1A shows the polished Ti surface before the HT treatment, and Fig. 1B displays the Ti surface after HT-TiO_2_ modification. After 2 h of mineralization (Fig. 1C), the HT-TiO_2_-coating was partially covered by Ca–P deposits. With extended mineralization times of 4 h and 6 h (Fig. 1D, E), the Ca-P coverage became more pronounced as confirmed by SEM-EDX (Fig. 1F). The mean CA measurements of the experimental groups (Fig. 1G) demonstrate that the NC group exhibited significantly higher mean CA values compared to all other groups (*p* < 0.001). Following HT-TiO2 coating, the mean CA was significantly reduced relative to the NC group (*p* < 0.001), and the subsequent RM treatment further decreased the mean CA values in a time-dependent manner, with the lowest value observed in the RM6 group (*p* < 0.001). The thickness of the Ca–P coating (Fig. 1H) increased significantly with increasing mineralization time from 2 to 6 h. The RM6 coating was significantly thicker than both RM4 (*p* < 0.001) and RM2 (*p* < 0.0001), and RM4 was significantly thicker than RM2 (*p* < 0.001).

#### Surface roughness

**Fig. 2:**
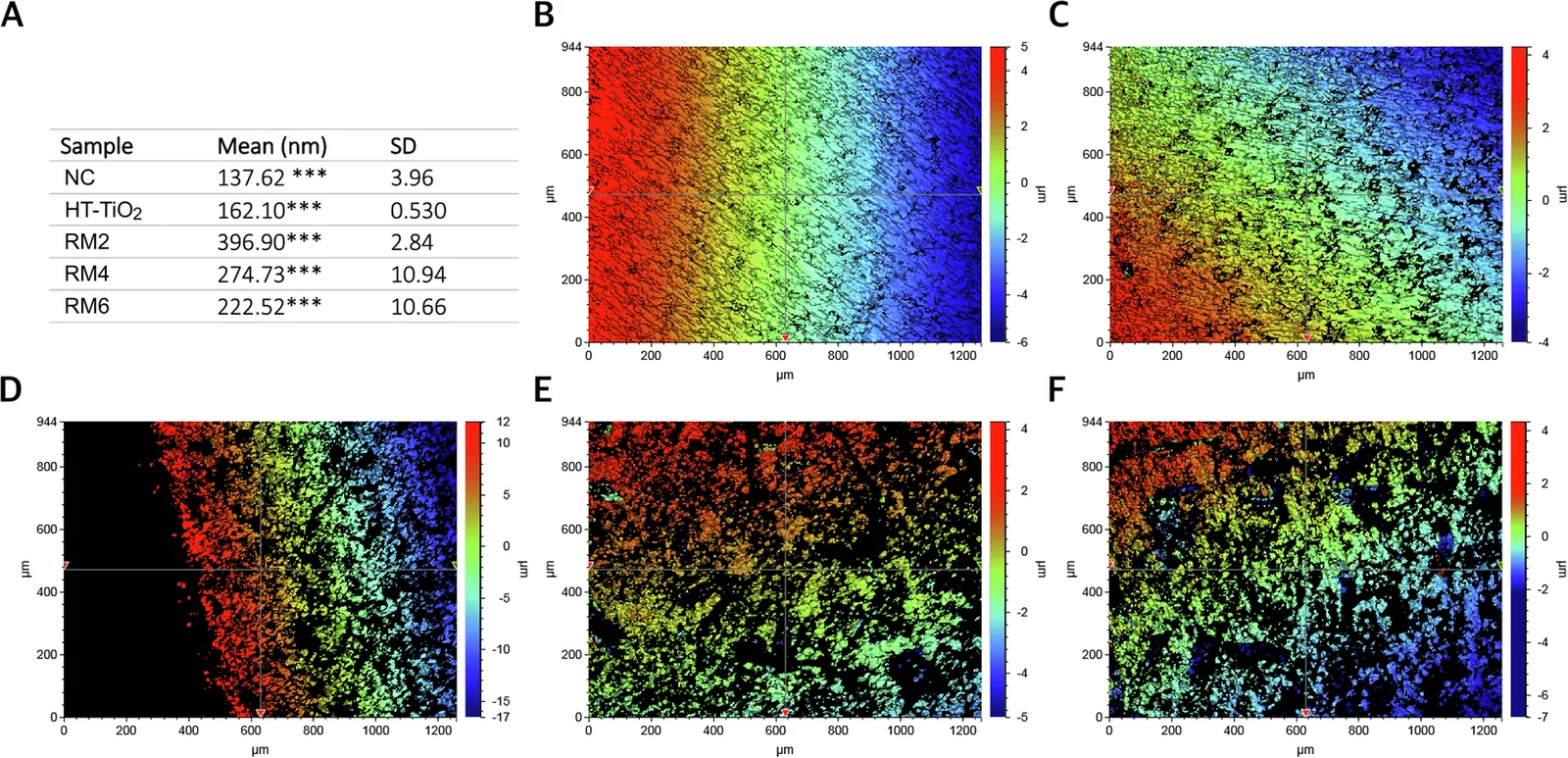
**A** Arithmetic mean height (Sa) and representative 3D surface profiles of the samples: **B** non-coated (NC), **C** hydrothermally induced nanostructured TiO_2_ (HT-TiO_2_), coating, and after rapid mineralization for **D** 2 h (RM2), **E** 4 h (RM4), and **F** 6 h (RM6). Images were acquired using a 5× objective lens with a 0.5× multiplier. The colors represent surface height variations, with warm colors such as red indicating peaks and cool colors such as blue indicating valleys. The black area in (**D**) corresponds to steep slopes with low reflectivity

The Sa surface roughness measurements (Fig. 2A) indicated significant differences among all experimental groups (*p* < 0.001), with the lowest Sa mean value observed for NC (137.61 ± 3.95 nm), followed by HT-TiO_2_ (162.09 ± 0.53 nm), RM6 (222.52 ± 10.65 nm), RM4 (274.72 ± 10.94 nm), and the highest value for RM2 (396.90 ± 2.84 nm). Figure 2B–F present 3D profilometer images illustrating surface height variations, with warm colors (red) indicating peaks and cool colors (blue) indicating valleys. The black area in Fig. 2D corresponds to steep slopes with low reflectivity.

#### X-ray diffraction pattern

**Fig. 3:**
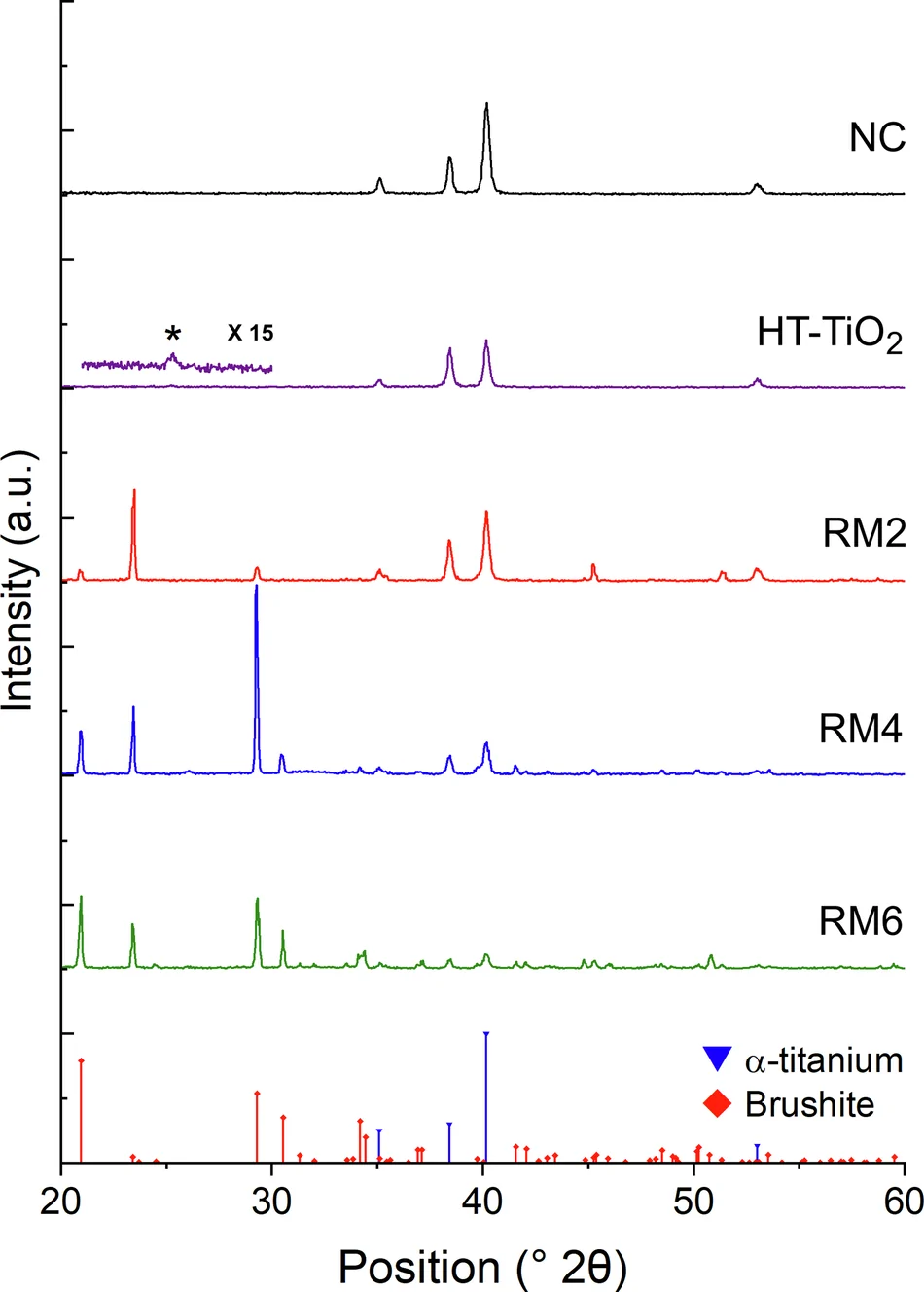
XRD diffractograms of the non-coated (NC) discs, hydrothermally induced nanostructured TiO_2_ (HT-TiO_2_) coatings and mineralization for 2 h (RM2), 4 h (RM4), 6 h (RM6), and the XRD brushite reference (PDF No. 01-072-0713). The TiO_2_ anatase phase is at 15X magnification

Figure 3 shows the grazing incidence (GIXRD) X-ray diffraction patterns of the NC, HT-TiO_2_, RM2, RM4, and RM6. The observed diffraction peaks were compared with standard Joint Committee on Powder Diffraction Standards (JCPDS) values to confirm the phase composition and formation. The NC surface exhibits distinct reflections corresponding to metallic Ti (JCPDS No. 00-044-1294), with prominent peaks observed at 2θ = 35.1°, 38.4°, 40.2°, and 53.0°. The HT-TiO_2_ surface demonstrated the presence of TiO_2_ anatase phase, indicated with an asterisk close to 25.3 degrees (JCPDS No. 03-065-5714). The other anatase peaks are mainly covered by the α -Ti peaks (37-38.5 degrees). Following mineralization, the diffraction patterns reveal characteristic reflections of brushite (CaHPO_4_ ∙ 2H_2_O), with peaks observed at 2θ = 11.6°, 21.0°, 23.4°, 29.3°, and 30.5°, indicating a substantial presence of brushite. Reference diffraction patterns for metallic Ti (JCPDS No. 00-044-1294) and brushite (JCPDS No. 01-072-0713) are shown in Fig. 3.

#### Fourier-transform infrared spectra

**Fig. 4:**
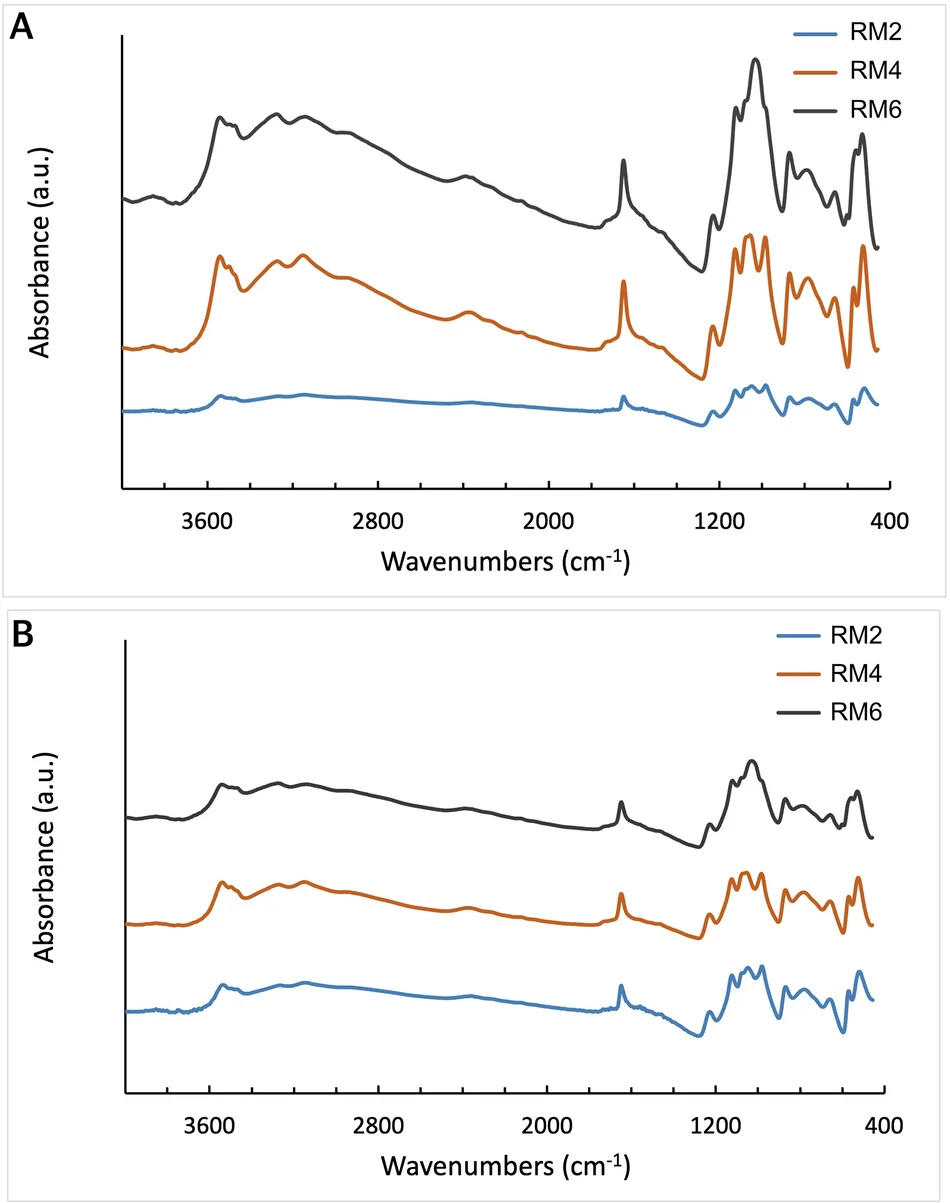
FTIR absorption spectra of mineralized hydrothermally induced nanostructured TiO_2_ (HT-TiO_2_) coated discs. **A** FTIR spectra of Ti discs after hydrothermally induced nanostructured TiO_2_ (HT-TiO_2_) coating and rapid mineralization for 2 h (RM2), 4 h (RM4), and 6 h (RM6). **B** Spectra were normalized to the total absorbance within the 457–1283 cm⁻¹ range to facilitate clearer comparison of spectral features and peak positions

Figure 4A shows the FTIR spectra of the RM2, RM4, and RM6 surfaces. To enable clearer comparison of spectral features and peak positions, the spectra in Fig. 4B were normalized to the total absorbance in the 457–1283 cm^−1^ range, where the most prominent vibrational bands appear. The mineralized surfaces exhibit characteristic features consistent with brushite [30, 31]. Absorptions attributed to P=O stretching vibrations are observed at 1218, 1120, and 1063 cm^−1^, while bands corresponding to P–O–P asymmetric stretching occur at 981, 869, and 786 cm^−1^ [32]. Additional vibrations at 659, 567, and 526 cm^−1^ are associated with acid phosphate (H–O–) P=O bond modes [33]. In the higher wavenumber region, bands at 3535, 3273, and 3145 cm^−1^ correspond to O–H stretching vibrations from structural water in brushite [34, 35]. A narrow peak at 1649 cm^−1^ is linked to H–O–H bending, while the broader absorption at 2379 cm^−1^ is related to HPO₄2- species. As shown in Fig. 4A, the intensity of the vibrational bands increases with longer mineralization durations, indicating progressive brushite deposition.

### Osteoblast proliferation and early osteogenic responses

Cellular responses to the NC, HT-TiO_2_, RM4, and RM6 Ti substrates were evaluated. The RM2 group was excluded from cellular experiments, as incomplete Ca-P coverage of the HT-TiO_2_ surface was observed, and its behavior was therefore expected to resemble that of the HT-TiO_2_ substrates.

#### Pre-osteoblast proliferation and live/dead analyses

**Fig. 5:**
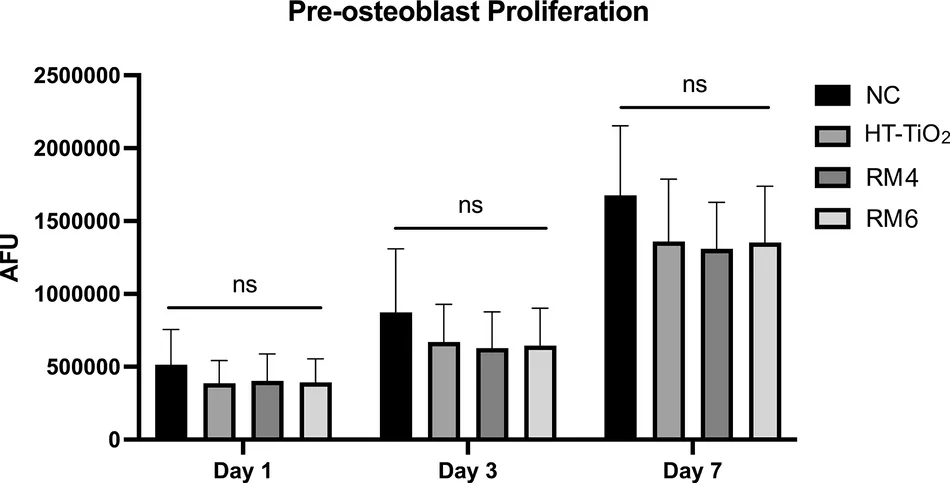
Cell proliferation of MC3T3-E1 cells cultured on non-coated (NC), hydrothermally induced nanostructured TiO_2_ (HT-TiO_2_) coating, and following mineralization for 4 h (RM4) and 6 h (RM6). Data are presented as arbitrary fluorescence units (AFU, 560/590 nm) on days 1, 3, and 7 of cell culture (*n* = 12). The data are presented as mean ± SD from three independent biological experiments, each performed with three replicates (*n* = 9)

**Fig. 6:**
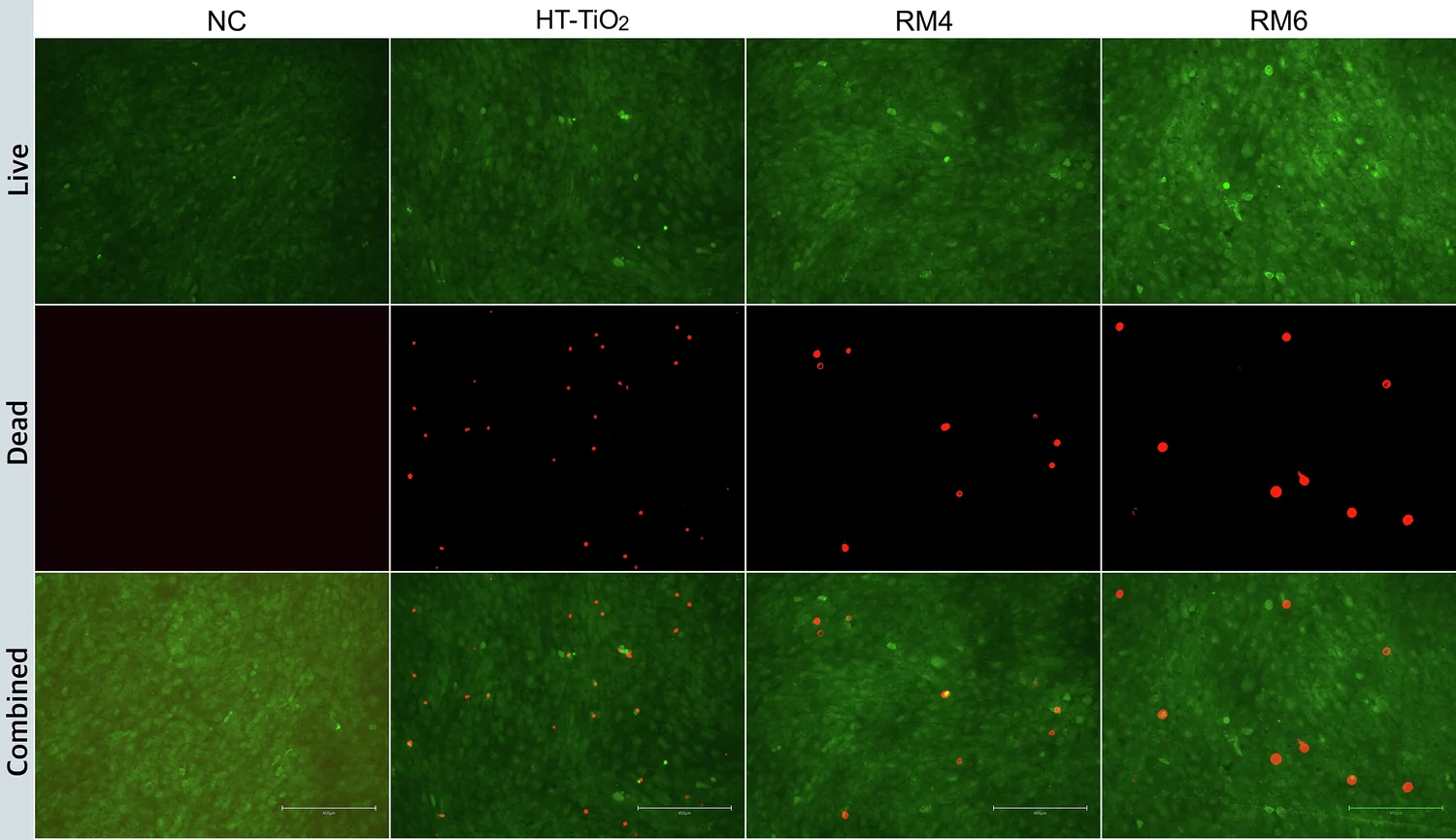
Live/Dead staining of MC3T3-E1 cells on non-coated (NC), hydrothermally induced nanostructured TiO_2_ (HT-TiO_2_) coating and following mineralization for 4 h (RM4) and 6 h (RM6) after 7 days of cell culture. Live cells are green and dead cells are red

All Ti discs supported the proliferation of pre-osteoblastic MC3T3-E1 cells, following a similar pattern across all experimental groups (Fig. 5). As the culture time extended, the cell numbers increased consistently. Even though the proliferation rates appeared slightly reduced in all experimental groups compared with NC Ti, no statistically significant differences were detected at any time point. Live/Dead staining at day 7 (Fig. 6) was consistent with the proliferation data. No dead cells were observed on NC Ti discs, while only a small number of dead cells were detected on the HT‑TiO₂, RM4, and RM6 surfaces.

#### DNA quantification

**Fig. 7:**
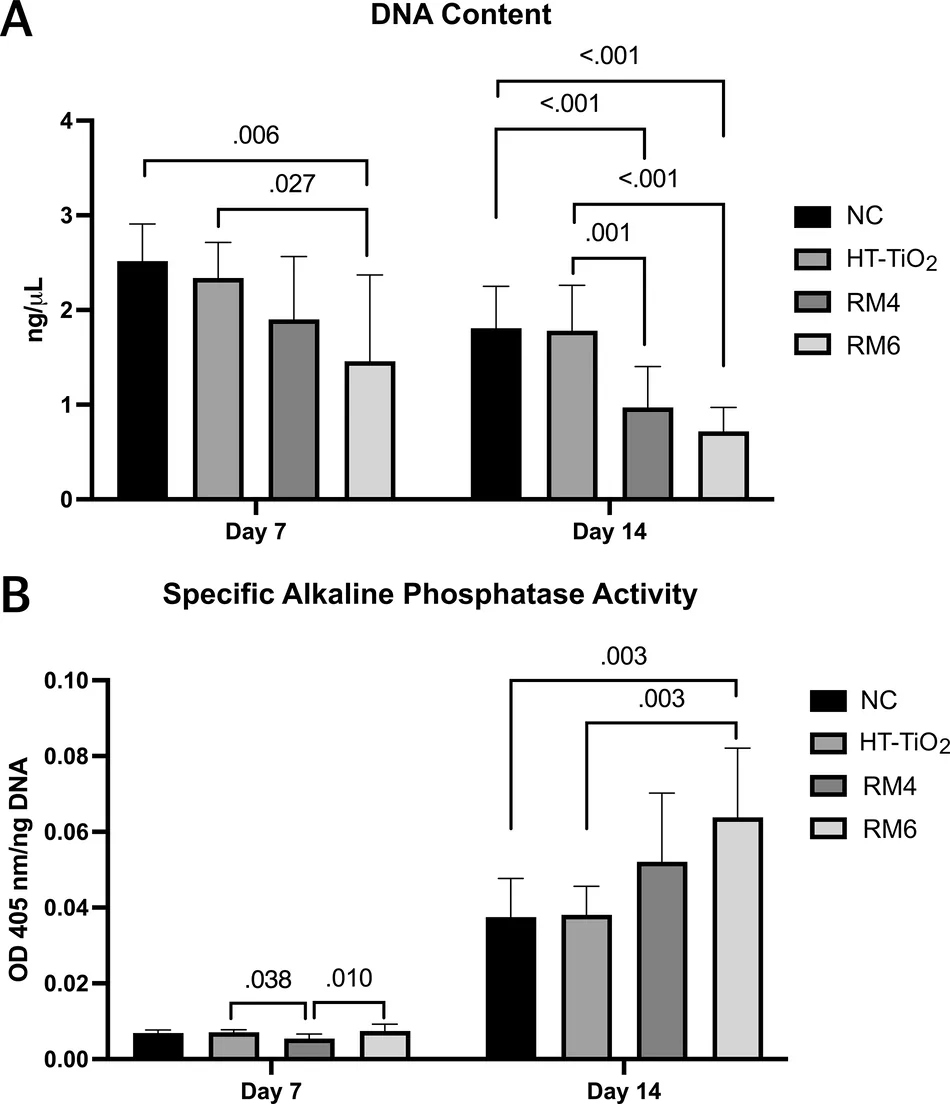
DNA content (**A**) and specific alkaline phosphatase (ALP) activity (**B**) of mesenchymal stem cell (MSC)-like cells cultured for 7 and 14 days on non-coated (NC), hydrothermally induced nanostructured TiO_2_ (HT-TiO_2_) coating and following mineralization for 4 h (RM4) and 6 h (RM6) hours. The data are presented as mean ± SD from three independent biological experiments, each performed with three replicates (*n* = 9)

DNA content, measured from the same lysates used for ALP activity assays to normalize enzymatic activity to cell number, revealed statistically significant differences between groups at days 7 and 14. At day 7, the NC (*p* = 0.006) and HT-TiO_2_ (*p* = 0.027) groups demonstrated significantly higher DNA content compared with the RM6 group. A similar pattern was observed at day 14, where the NC and HT-TiO_2_ groups showed significantly higher DNA content (*p* < 0.001) than both the RM4 and RM6 groups (Fig. 7A).

#### ALP activity and osteogenesis-related staining of MSC-like cells

The osteogenic induction capacity of C3H10T1/2 MSC‑like cells was verified by ALP and von Kossa staining performed on cells cultured on tissue‑culture plastic under basal or osteogenic conditions. As shown in Supplementary Fig. 1, cells maintained in osteogenic media exhibited positive ALP staining and mineral deposition, whereas cells cultures in basal media showed no positive staining. However, within the osteogenic cultures, morphological heterogeneity was observed among MSC-like cells, with some cells displaying a rounded morphology with intracellular vacuoles resembling adipocyte‑like cells (Supplementary Fig. S1). These cells did not exhibit ALP activity, indicating the presence of distinct cellular subpopulations within the cultures.

Quantitative ALP activity was assessed for MSC-like cells cultured on Ti substrates at days 7 and 14 (Fig. 7B). ALP activity was significantly higher in the HT-TiO_2_ group (*p* = 0.038) and RM6 group (*p* = 0.010) compared to the RM4 group, while no significant differences were observed relative to the NC control. By day 14, the RM6 group exhibited significantly higher ALP activity than the NC and HT-TiO_2_ groups (*p* = 0.003), but did not significantly differ from the RM4 group (Fig. 7B).

#### Attachment, spreading, and morphology of MSC-like cells

**Fig. 8:**
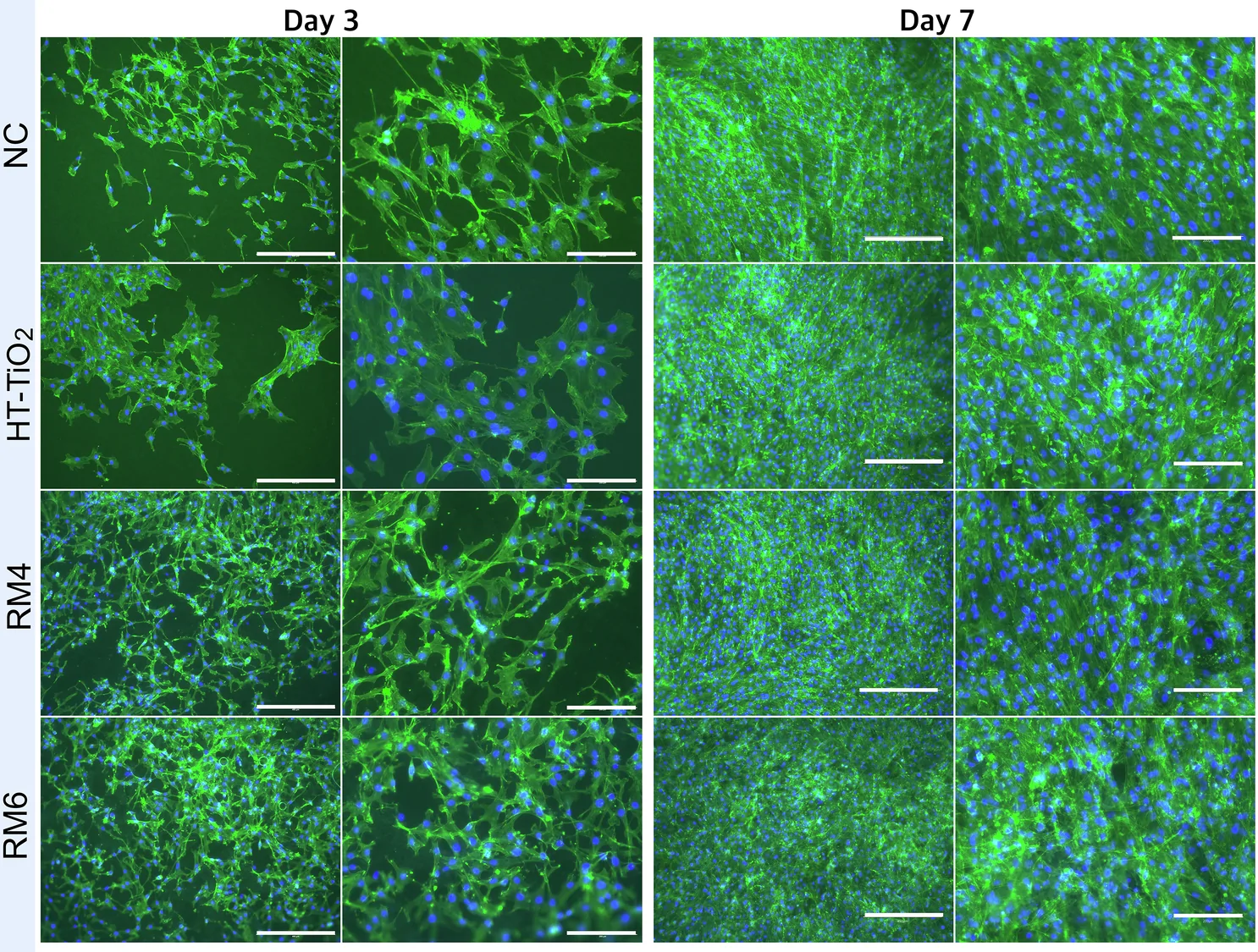
Fluorescence microscope images of the attachment and spreading of mesenchymal stem cell (MSC)-like cells cultured for 3 and 7 days on non-coated (NC), hydrothermally induced nanostructured TiO_2_ (HT-TiO_2_) coating, and following mineralization for 4 h (RM4) and 6 h (RM6). Cytoskeletal Actin is stained green, while nuclei are blue. Magnifications (10X; scale bar 450 μm and 20X; scale bar 200 μm) are indicated in images

As shown in Fig. 8, C3H10T1/2 MSC-like cells cultured on HT-TiO_2_ discs exhibited a flattened cytoskeletal morphology at day 3. In contrast, cells on mineralized Ti discs (RM4 and RM6) showed a more elongated, spindle-shaped morphology with multiple cellular protrusions. By day 7, however, all discs were extensively covered by MSC-like cells, with comparable levels of confluency and a more uniformly organized cell layer observed across all groups.

## Discussion

This study demonstrates that rapid mineralization of HT-TiO_2_-coating can support osteoblast viability and proliferation, while modulating early cellular responses at the material surface. Compared with shorter mineralization times, a six-hour mineralization period resulted in the highest surface Ca and P content on the HT-TiO_2_ coating and increased ALP activity. Nevertheless, this increase should be interpreted as early osteogenic activity in a subset of MSC‑like cells, rather than as evidence of enhanced or definitive osteogenic differentiation.

To assess the surface biocompatibility and early biological responses, cell proliferation and ALP activity assays were conducted on NC, HT-TiO_2_, RM4, and RM6 substrates. The slightly reduced proliferation rates and the presence of a small number of non-viable cells observed in the HT-TiO_2_ and RM groups compared with the NC group may reflect altered surface chemistry or local cellular stress responses associated with the mild oxidative stress induced by the TiO_2_ layer [36], rather than cytotoxic effects, as overall cell viability remained high.

Alkaline phosphatase (ALP) is the key enzyme involved in bone formation and is widely used as an early marker of osteogenic activity [37]. In the present study, ALP activity in MSC-like cells was significantly higher in the RM6 group at day 14 compared to the NC and HT-TiO_2_ groups, although no significant difference was observed when compared with the RM4 group. Nevertheless, while the RM4 group also showed higher ALP activity than NC and HT-TiO_2_ groups, the differences between mineralized groups highlight a graded surface-dependent reponse rather than uniform osteogenic differentiation.

The elevated ALP activity observed on Ca-P-containing surfaces suggests that increased surface Ca availability can modulate early osteogenic activity in MSC-like cells, consistent with previous studies on the role of calcium in osteogenic responses at biomaterial interphases [38]. Calcium has also been shown to influence early osteogenic signaling, including non-canonical Wnt/Ca^2+^ pathways [39–41], and to function as a paracrine chemotactic factor, facilitating the migration of mesenchymal precursor cells [42]. However, such responses could also reflect early cellular activity rather than definitive lineage commitment.

In this study, ALP and von Kossa staining were performed solely to verify the osteogenic induction capacity of the MSC‑like cell line under osteogenic culture conditions on tissue‑culture plastic. These stainings confirmed the presence of ALP‑positive cells and mineral deposition in osteogenic media but also revealed morphological heterogeneity within the cultures, underscoring the multipotent nature of the C3H10T1/2 cell line.

During osteogenic induction, MSCs typically exhibit a progressive decline in proliferative capacity due to arrested self-renewal, which is subsequently accompanied by increased expression of early osteogenic markers, such as ALP. The observed time-dependent decrease in DNA content is consistent with alterations in cellular state under osteogenic induction conditions [43–46], but should not be interpreted as evidence of uniform osteogenic differentiation, in which cell division typically slows as differentiation-related activities become more pronounced. While ALP remains a useful early marker of osteogenic activity, it is not specific to late/mid osteogenic differentiation or matrix mineralization. Additional markers, such as Runx2, collagen type I, osteopontin and osteocalcin expression, would provide more comprehensive insights into definitive osteoblastic maturation [47]. Accordingly, the present findings should be interpreted as indicative of early osteogenic activity and heterogeneous cellular responses, rather than conclusive evidence of complete osteoblastic differentiation.

Surface properties are well documented to play a crucial role in modulating cell attachment, spreading, and morphology [48–50]. Fluorescence microscopy imaging of MSC-like cells at day 3, following staining for nuclei and F-actin, revealed distinct morphological differences between cells cultured on HT-TiO_2_ discs compared to cells on RM discs, indicating substrate-dependent variations in cytoskeletal organization and cell spreading. Cells cultured on HT-TiO_2_-coated discs exhibited a more flattened morphology, whereas those on the RM4 and RM6 discs predominantly displayed an elongated, spindle-like shape with multiple protrusions.

Such morphological differences are likely influenced by variations in surface topography. Before mineralization, HT-TiO_2_-coated discs presented a relatively flat and uniform surface, which may have facilitated wider cell spreading. In contrast, RM treatment resulted in a more porous, rough, and irregular surface, likely guiding cells toward a spindle-like morphology. Consistent with this observation, previous studies have reported that surface micropatterning and increased roughness can reduce cell spreading [43–45]. The protrusions observed on MSC-like cells cultured on RM discs may reflect characteristic features associated with osteoblast-like morphology, as differentiating osteoblasts are known to extend cellular protrusions into adjacent mineral regions during matrix formation [51]. Moreover, cell size and shape, largely regulated by F-actin dynamics, play a pivotal role in directing downstream processes, including osteogenic activity [51]. Indeed, MSCs grown on porous surfaces or within confined geometries have been reported to exhibit earlier and more pronounced osteogenic commitment [52]. In the present study, no clear morphological differences were observed between groups by day 7, as cells across all surfaces had reached similar levels of confluency and spreading. This uniformity in morphology is likely associated with their proliferation dynamics, which typically peak around this time point. Under sub-confluent conditions, cells display greater motility and spreading due to available surface area, whereas increasing confluency imposes spatial constraints that promote a more compact morphology. As culture time progresses, cytoskeletal reorganization occurs to accommodate higher cell density and promote further spreading [53, 54]. The uniform confluency observed across all groups confirms the biocompatibility of the investigated surfaces in supporting MSC-like cell attachment and growth.

Surface roughness is a critical factor affecting osteoblast proliferation and differentiation. Moderate roughness values (100–200 nm) have been shown to favor osteoblast differentiation, whereas surfaces with either very low or very high roughness appear to be less supportive to osteoblast function [55, 56]. In the present study, the NC group displayed the lowest average surface roughness (Sa) values among all experimental groups. Although no statistically significant differences in pre-osteoblast proliferation were observed, the NC group showed the highest proliferation levels overall.

Rapid mineralization (RM) of HT-TiO_2_-coated discs initially increased surface roughness; however, Sa values decreased with increasing mineralization time. This trend likely reflects the progressive deposition of Ca-P on the surface: at 2 h, incomplete coverage resulted in irregular pores and a rougher surface, whereas extended mineralization times of 4 and 6 h produced more uniform Ca–P layers, likely leading to smoother topographies. Despite the NC and HT-TiO_2_ groups having significantly smoother surfaces, ALP activity was highest in the RM6 group. This response may be attributed to the combined effects of Ca–P composition and a relatively favorable surface roughness [57].

The XRD analysis of the RM surfaces showed variations in peak intensities and relative peak ratios across different mineralization times, suggesting changes in the microstructure of the deposited brushite layer, such as preferred crystal orientation. The grazing incidence geometry may contribute to these variations by enhancing surface sensitivity. In addition to reflections from the deposited layer, peaks from the underlying Ti substrate were still detectable, though their intensities gradually decreased with longer mineralization times. After 6 h, these substrate peaks were nearly absent, indicating more complete surface coverage and/or increased layer thickness. These trends were supported by SEM-EDX observations, which showed a corresponding increase in the surface coverage and in the molar composition of Ca and P with longer mineralization times.

Surface wettability is another key factor, typically assessed through CA measurements, influencing protein adsorption, which in turn is known to play a critical role in regulating osteoblast proliferation and differentiation [56, 58–61]. In particular, lower CA values, corresponding to more hydrophilic surfaces, have been reported to enhance osteoblast attachment [62]. In the present study, all tested surfaces displayed a CA below 90° and can thus be classified as hydrophilic. Among the tested groups, the measured CAs were 81.50° for NC, 72.08° for HT-TiO_2_, 34.69° for RM4, and 24.30° for RM6, showing a disproportional relationship with surface roughness.

The observed variations in hydrophilic behavior may be related to differing combinations of microtopography and surface chemistry, as wettability is influenced by both the chemical composition and the physical structure of the surface. Studies have shown that the Ca-P coatings result in lower CA values [63], likely due to enhanced water adsorption onto the coating surface [64]. Despite these differences in surface wettability, no statistically significant differences in pre-osteoblast proliferation were observed among the groups at any time point, suggesting that while surface properties may influence early cell attachment and morphology, they do not necessarily impact proliferation under the tested conditions. On the other hand, it has been reported that highly hydrophilic surfaces can promote osteogenic activity and differentiation-related responses in MSC-like cells, particularly under osteogenic induction conditions [65].

A previous study investigated the mineralization of Ti strips using a Ca–P solution of similar concentration to that used in the present work. The study reported coating adhesion strengths of ~12 MPa, comparable to those achieved with coatings formed by immersion in 1.5× SBF over 14 days [23]. These findings suggest that the Ca–P coating produced in the current study may possess similar mechanical stability, although further verification is required. Brushite, which is also known as dicalcium phosphate dihydrate (DCPD), has a Ca/P ratio of 1 [66], whereas the Ca/P ratio of HA is 1.67. Brushite can exist as an intermediate phase during the precipitation of HA and bone mineralization [67, 68]. In this study, the Ca/P ratios obtained were 0.93, 1.19, and 1.13 for RM2, RM4, and RM6, respectively. The ratio of the RM2 group may suggest a very early amorphous Ca-P; therefore, it was another reason to exclude it from the cell study. Brushite has a faster solubility rate in physiologic conditions compared to HA. However, this may have the advantage of the controlled release of Ca and P ions, which are essential for the mineralization of bone tissue [69]. Furthermore, brushite can adsorb biological molecules, making it an ideal scaffold for cell growth and the formation of new bone tissue [69, 70]. However, in the current study, the solubility and the HA transformation of the brushite were not investigated.

Osseointegration of dental and orthopedic implants is a complex biological process influenced by bone quality (e.g., osteoporosis) [71, 72], host immunity, metabolic status (e.g., diabetes mellitus) [73], as well as patient-related factors such as aging [74]. These patient groups often exhibit reduced bone mineral density, which may lead to delayed bone formation and remodeling. Such delays can impair bone-to-implant contact and ultimately increase the risk of implant failure. The rapid mineralization of the HT-TiO₂ coating achieved in this study, formed on Ti surfaces within a relatively short time, represents a key advantage over conventional surface modification approaches that require prolonged processing times. This rapidly formed Ca-P layer may serve as an additional local source of Ca and P ions, supporting early mineral interactions at the implant interface and potentially enhancing the initial healing of the mineralized tissue surrounding the implant. Importantly, the modified surfaces were shown to support osteoblast viability and proliferation and to modulate early osteogenic activity, which together may contribute to improved osseointegration, particularly in conditions with compromised bone quality. However, the release kinetics of Ca and P ions from the coating were not assessed in the present study and should be addressed in future work to better understand their contribution to the biological response.

In the current study, a modified Ti surface with bioactive properties was developed using a simple and rapid processing strategy, highlighting its potential for translational application. Future research may focus on functionalizing the coating with antibacterial properties by the incorporation of bioactive ions, such as zinc and strontium, into the Ca–P layer. While in vitro studies provide valuable insights into early cell–material interactions and surface-driven cellular responses, they cannot fully recapitulate the complexity of the in vivo environment, where factors such as immune modulation, vascularization, and mechanical loading play important roles. Consequently, further in vivo investigations are required to establish the clinical relevance and long‑term performance of the proposed surface modification strategy.

## Conclusions

Hydrothermally-induced nanostructured TiO_2_ coatings on titanium surfaces can be rapidly mineralized in a supersaturated simulated body fluid, resulting in the formation of a calcium–phosphate layer in a time-dependent manner. An extended mineralization period of 6 h yields a more continuous and compositionally developed mineralized layer. The mineralized surfaces supported osteoblast viability and proliferation and were associated with increased alkaline phosphatase (ALP) activity in a subset of mesenchymal stem cell-like cells. These findings indicate modulation of early osteogenic activity, rather than definitive osteogenic differentiation. Overall, the results highlight the potential of rapid, surface‑driven mineralization strategies to influence early cell-material interactions relevant to implant osseointegration, while highlighting the need for further studies to assess ion release dynamics and in vivo performance.

## Supplementary information

Supplementary information

Supplementary information

## Data Availability

The data that support the findings of this study are available from the corresponding author upon reasonable request.
