# Supplementary material for: Rapid mineralization of hydrothermally induced TiO2 coating on titanium and early osteogenic responses in vitro
Source: J Mater Sci Mater Med. 2026 Jun 16;37(1):108. doi: 10.1007/s10856-026-07095-2 (PMC13506634; doi:10.1007/s10856-026-07095-2)
Supplement: Supplementary file 1 [file 10856_2026_7095_MOESM2_ESM.docx]

**Supplementary Figure 1.** ALP and von Kossa staining of MSC‑like cells under basal and osteogenic conditions. Representative images of alkaline phosphatase (ALP) staining and von Kossa staining of C3H10T1/2 MSC‑like cells cultured on tissue‑culture plastic under basal medium or osteogenic induction medium at day 7. Images are shown for qualitative visualization only and magnification magnification (ALP 10X; scale bars 450 μm and von Kossa 4X; scale bar 800 μm) is indicated in images.
